# High expression of microRNA-454 is associated with poor prognosis in triple-negative breast cancer

**DOI:** 10.18632/oncotarget.11764

**Published:** 2016-08-31

**Authors:** Zhi-Gang Cao, Jun-Jing Li, Ling Yao, Yan-Ni Huang, Yi-Rong Liu, Xin Hu, Chuan-Gui Song, Zhi-Ming Shao

**Affiliations:** ^1^ Department of Breast Surgery, Key Laboratory of Breast Cancer in Shanghai, Fudan University Shanghai Cancer Center, Shanghai, China; ^2^ Department of Breast Surgery, Affiliated Union Hospital, Fujian Medical University, Fuzhou, China

**Keywords:** microRNA-454, triple-negative breast cancer, disease-free survival, chemotherapy, in situ hybridization

## Abstract

MicroRNA-454 (miR-454) has been reported to play an oncogenic or tumor suppressor role in most cancers. However, the clinical relevance of miR-454 in breast cancer remains unclear. We examined the expression of miR-454 in a tissue microarray containing 534 breast cancer specimens from female patients at Fudan University Shanghai Cancer Center using *in situ* hybridization (ISH). Of these, 250 patients formed the training set and the other 284 were the validation set. The relationship between miR-454 and clinical outcome was analyzed by the Kaplan-Meier method. High expression of miR-454 indicated worse disease-free survival (DFS) in both cohorts (*P* = 0.006 for training set; *P* = 0.010 for validation set). Furthermore, in the triple-negative breast cancer (TNBC) subtype, miR-454 was positively correlated with worse clinical outcome (*P* = 0.013 for training set, *P* = 0.014 for validation set). In addition, patients in the low miR-454 expression cohort had better response to anthracycline compared to non-anthracycline chemotherapy (*P* = 0.056), but this difference was not observed in the high miR-454 expression cohort. Our findings indicated that miR-454 is a potential predictor of prognosis and chemotherapy response in TNBC.

## INTRODUCTION

Breast cancer is the most frequently diagnosed cancer and the leading cause of cancer death among females worldwide, with an estimated 1.7 million cases and 521,900 deaths in 2012 [1]. Breast cancer can be classified into four different molecular subtypes, according to hormone receptor and human epidermal growth factor receptor type 2 (HER2) status, as follows: Luminal A, Luminal B, HER2/neu+, and triple-negative [2]. Triple-negative breast cancers are defined as tumors that lack expression of estrogen receptor (ER), progesterone receptor (PR), and HER2, and patients with triple-negative tumors have a relatively poor outcome [3]. Because of their specific receptor status, patients with triple-negative breast cancer do not benefit from endocrine therapy or trastuzumab [4]. Therefore, chemotherapy is currently the mainstay of systemic treatment.

MicroRNAs (miRNAs) are endogenous RNAs of approximately 23 nucleotides that can play important gene regulatory roles by pairing to the mRNAs of protein-coding genes and directing their post-transcriptional repression [5]. Numerous studies have shown that miR-454 expression correlates with tumor growth, invasion, and metastasis in many cancers; however, miR-454 has a dual function, and can act as an oncogenic miRNA or a tumor suppressor. In hepatocellular carcinoma, knockdown of miR-454 inhibited HCC cell proliferation, and invasion and EMT [6], and miR-454 was proposed be a valuable prognostic factor [7]. Overexpression of miR-454 resulted in significant promotion of cell proliferation, colony formation, invasion, and induction of cell cycle in uveal melanoma cells [8]. The prognosis of glioma with high miR-454 expression was significantly worse than that of glioma with low miR-454 expression, suggesting that miR-454 could be a novel potential prognosis biomarker [9]. miR-454 is downregulated in esophageal cancer compared with paracancerous normal tissues [10], and acts as a tumor suppressor in osteosarcoma [11]. In renal carcinoma cells, overexpression of miR-454 enhanced radiosensitivity and shifted the cell cycle [12]. However, few studies have evaluated the relationship between miR-454 and clinical outcome in breast cancer. One study reported that miR-454-3p was upregulated in early-stage breast cancer [13]. Also, in Hispanic women, miR-454 was overexpressed in the group of women diagnosed with breast cancer < 5.2 years postpartum compared with the late diagnosis group [14].

Although there were some reports of miR-454 expression in breast tumors, we still know little about the predictive role of miR-454 in breast cancer. The aim of this study was to investigate the value of miR-454 for predicting the clinical outcome of breast cancer. We examined miR-454 expression in breast cancer specimens of two patient cohorts (a total of 534 patients) by ISH, and evaluated the correlation of miR-454 expression and disease-free survival (DFS) or overall survival (OS).

As mentioned above, chemotherapy is a crucial treatment modality for TNBC and numerous large randomized trials have established the benefit of adjuvant anthracyclines and taxanes in breast cancer [15]. Dysregulation of miRNAs in breast cancer be involved in the response and resistance to chemotherapy [16-18], and can therefore serve as predictive biomarkers of therapeutic outcomes [19]. Therefore, we also analyzed the role of miR-454 in predicting the response to different chemotherapeutic agents.

## RESULTS

### Clinical characteristics of patients and miR-454 expression pattern

**Figure 1:**
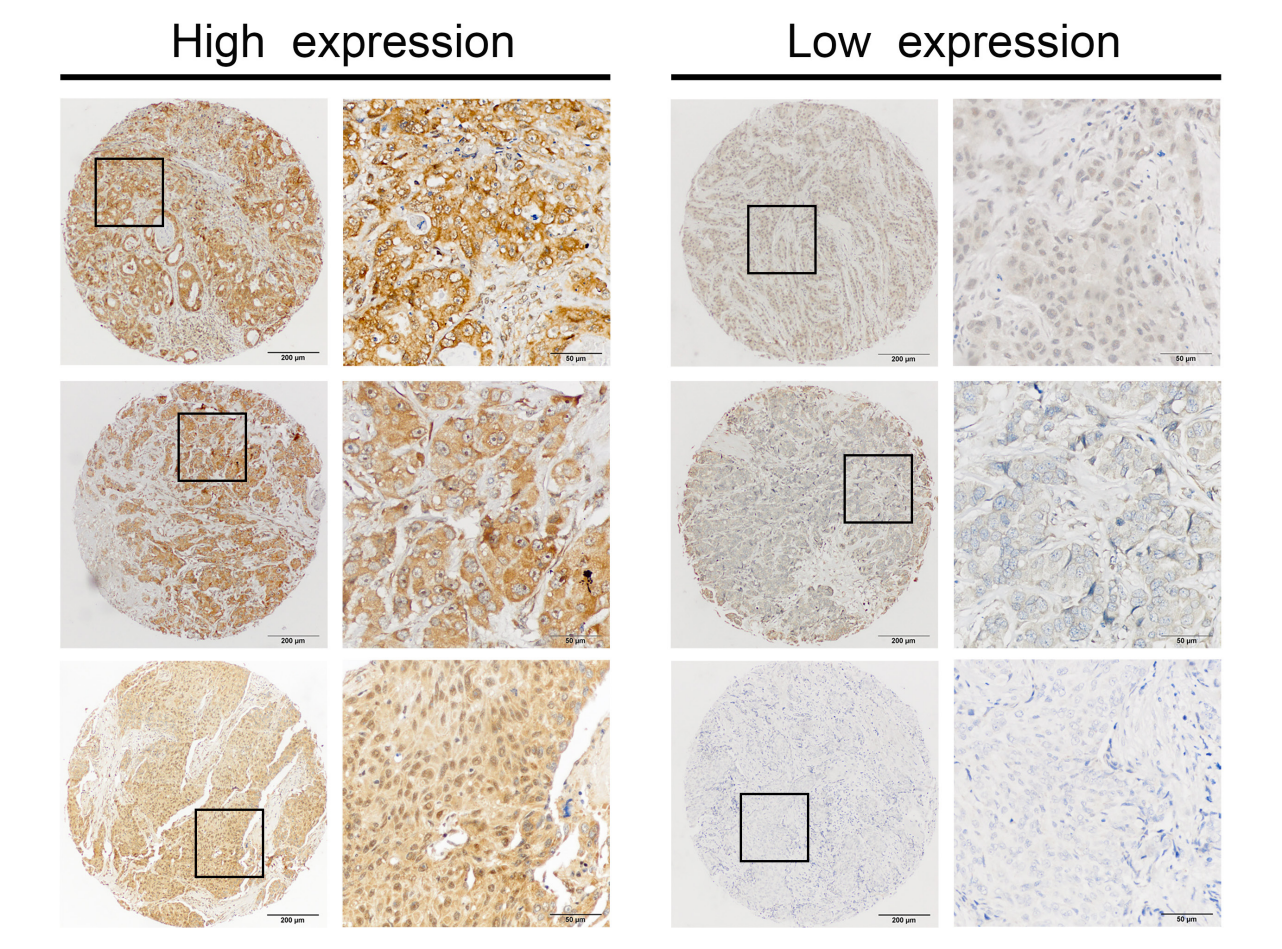
Identification of miR-454 in primary tumors by *in situ* hybridization (ISH) Representative IHC staining of high and low miR-454 expression is presented at 400× magnification and 100× magnification.

The training set included a total of 250 female breast cancer patients. Of these, 25 cases lacked follow-up data or tissue cylinders were lost after ISH. The median age of the remaining 225 participants was 50 years (inter-quartile range [IQR] 45-56 years) at the time of surgery, and recurrence or death occurred in 53 of 225 cases after a median follow-up time of 94.50 months (IQR 57.75-117.47 months). In the validation set, 43 cases that either lacked follow-up data or experienced tissue loss after ISH staining were excluded, and the median age of the remaining 241 patients was 50 years (IQR 44-58 years) and median follow-up time was 80.40 months (IQR 35.54-98.50 months). A total of 55 patients in cohort 2 experienced distant metastasis or death. As shown in Table S1, there was no significant difference in clinical characteristics of the two cohorts. We used the miRCURY LNA^TM^ detection probe labeled at the 5′-end and 3′-end with digoxigenin to specific examine expression and reveal the clinical significance of miR-454 in TMAs using ISH. Representative images of miR-454 expression are presented in Figure 1. As described in Table 1, positive staining for miR-454 was observed in 36.0% (81/225) of the training set and 36.5% (88/241) of the validation set using the criterion described above. The correlations between patients' clinical characteristics and levels of miR-454 are summarized in Table 1. No significant associations were observed between miR-454 expression and clinicopathologic characteristics.

**Table 1:** Clinicopathological variables and the expression of miR-454 in the training set and validation set

Variable	Training set				Validation set			
	Number	miR-454 expression		*P^a^*	Number	miR-454 expression		*P^a^*
		Low N (%)	High N (%)			Low N (%)	High N (%)	
Total	225	144(64.0%)	81(36.0%)		241	153(63.5%)	88(36.5%)	
Age				0.296				0.525
≤50years	116	78(54.2%)	38(46.9%)		127	83(54.2%)	44(50.0%)	
>50 years	109	66(45.8%)	43(53.1%)		114	70(45.8%)	44(50.1%)	
Menstrual status				0.470				0.518
Premenopause	110	73(50.7%)	37(45.7%)		138	90(58.8%)	48(54.5%)	
Postmenopause	115	71(49.3%)	44(54.3%)		103	63(41.2%)	40(45.5%)	
Grade				0.337				0.143
1 or 2	127	86(59.7%)	41(50.6%)		120	79(51.6%)	41(46.6%)	
3	60	34(23.6%)	26(32.1%)		81	45(29.4%)	36(40.9%)	
Unknown	38	24(16.7%)	14(17.3%)		40	29(19.0%)	11(12.5%)	
Tumor size				0.309				0.542
≤2 cm	109	75(52.1%)	34(42.0%)		100	60(39.2%)	40(45.5%)	
>2,≤5 cm	105	61(42.4%)	44(54.3%)		121	80(52.3%)	41(46.6%)	
>5cm	9	7(4.9%)	2(2.5%)		14	8(5.2%)	6(6.8%)	
Not measurable	2	1(0.7%)	1(1.2%)		6	5(3.3%)	1(1.1%)	
Lymph node status				0.114				0.318
Negative	128	87(60.4%)	41(50.6%)		135	82(53.6%)	53(60.2%)	
Positive	97	57(39.6%)	40(49.4%)		106	71(46.4%)	35(39.8%)	
ER status				0.361				0.850
Negative	144	89(61.8%)	55(67.9%)		157	99(64.7%)	58(65.9%)	
Positive	81	55(38.2%)	26(32.1%)		84	54(35.3%)	30(34.1%)	
PR status				0.323				0.470
Negative	159	105(72.9%)	54(66.7%)		179	116(75.8%)	63(71.6%)	
Positive	66	39(27.1%)	27(33.3%)		62	37(24.2%)	25(28.4%)	
Her-2 status				0.130				0.905
Negative	137	93(64.6%)	44(54.3%)		160	102(66.7%)	58(65.9%)	
Positive	88	51(35.4%)	37(45.7%)		81	51(33.3%)	30(34.1%)	
Molecular Subtype^b^				0.195				0.843
Luminal A	44	33(22.9%)	11(13.6%)		44	30(19.6%)	14(15.9%)	
Luminal B	47	25(17.4%)	22(27.2%)		40	24(15.7%)	16(18.2%)	
HER-2+	42	27(18.8%)	15(18.5%)		41	27(17.6%)	14(15.9%)	
Triple-negative	92	59(41.0%)	34(40.7%)		116	72(47.1%)	44(50.0%)	

Abbreviations: IQR, inter-quartile range; ER, estrogen receptor; PR, progesterone receptor; HER-2, human epidermal growth factor receptor 2.

a Based on the Pearson χ2 test (Fisher's exact test was used when needed).

b Definitions of subtypes: Luminal A (ER- and/or PR-positive, HER-2-negative, PR high expression and Ki-67 low expression), Luminal B (ER- and/or PR-positive, HER-2-positive; ER-and/or PR-positive, HER-2-negative and Ki-67 high expression or PR low expression), HER-2+ (ER- and PR-negative, HER-2-positive), and Triple-negative (ER-negative, PR-negative, and HER-2-negative).

### The relationship between the expression of miR-454 and clinical outcome in breast cancer patients

**Figure 2:**
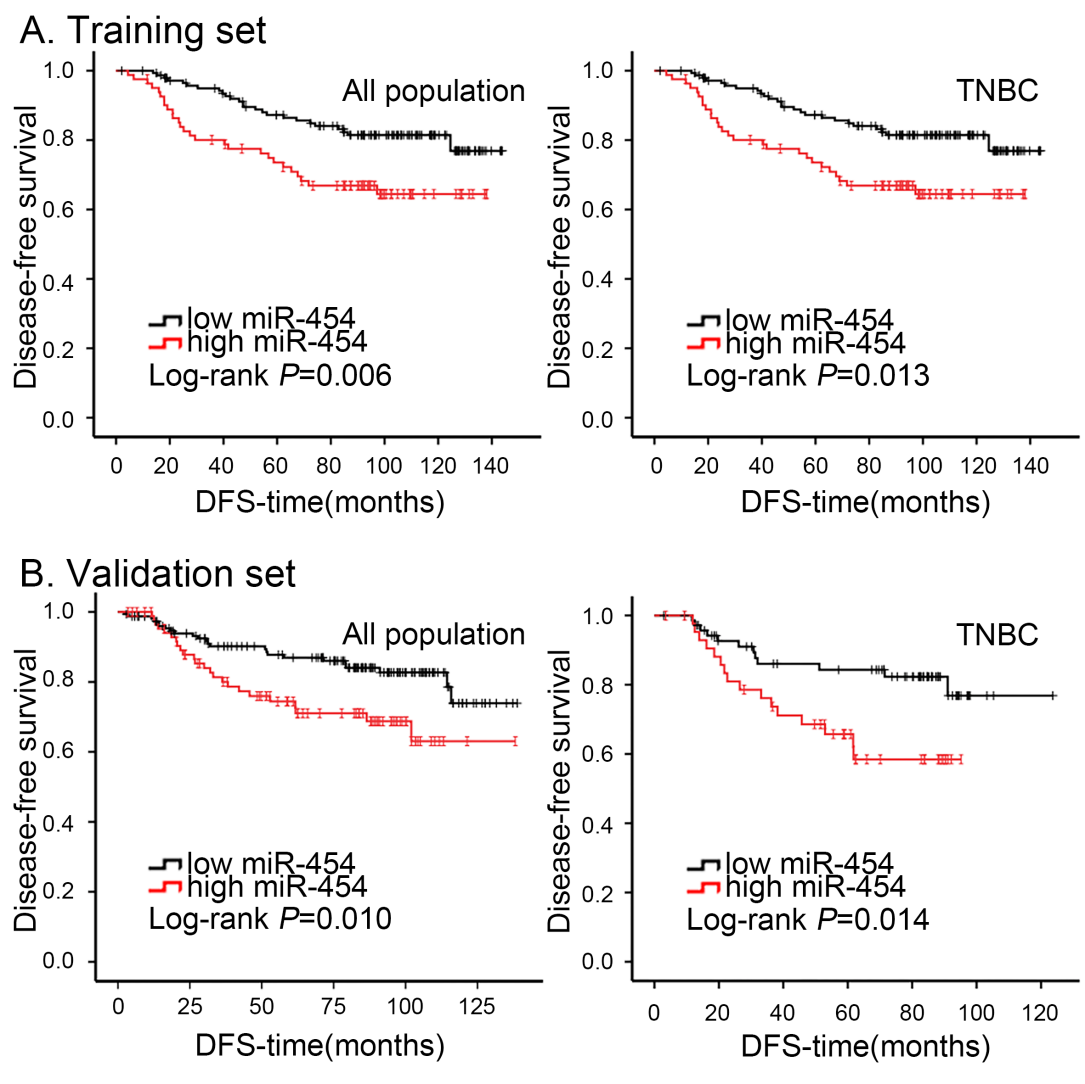
Kaplan-Meier analysis of DFS in breast cancer patients with high or low miR-454 expression **A.** Cumulative DFS curves of breast cancer patients with high or low miR-454 expression in overall population and TNBC subtype of training set. **B.** Cumulative DFS curves of breast cancer patients with high or low miR-454 expression in overall population and TNBC subtype of validation set.

We evaluated the correlation between miR-454 status and clinical outcome to evaluate the clinical significance of this miRNA in breast cancer. In both sets, Kaplan-Meier analysis showed that DFS was significantly worse in the high miR-454 expression group than in the low miR-454 expression group (*P* = 0.006 for training set, Figure 2A; *P* = 0.010 for validation set, Figure 2B). To further investigate the association of miR-454 with prognosis, we performed stratification of the breast tumors by hormone receptor and HER2 status. Elevated expression of miR-454 correlated with favorable DFS in the TNBC group with statistical significance (*P* = 0.013 for training set, Figure 2A; *P* = 0.014 for validation set, Figure 2B), but not in other subtypes (Figure S1). Notably, similar associations between miR-454 with OS were identified in the TNBC subtype (Figure S2). In conclusion, high expression of miR-454 indicated a worse clinical outcome in the overall population and the TNBC subtype.

### Univariate and multivariate analyses of prognostic factors in overall breast cancer populations and TNBC

As shown in Table 2, in the overall breast cancer population the results of univariate analyses indicated that overexpression of miR-454 expression was associated with a higher risk of recurrence (HR = 2.10, 95% CI 1.22-3.60, *P* = 0.007 for training set; HR = 2.08, 95% CI 1.18-3.69, *P* = 0.012 for validation set). Multivariate analysis considering the relationship between clinical factors revealed that high expression of miR-454 was independently associated with a worse DFS (HR = 2.02, 95% CI 1.10-3.72, *P* = 0.023 for training set; HR = 2.16, 95% CI 1.16-4.03, *P* = 0.015 for validation set). Tumor size > 5 cm and positive lymph node status were also associated with poor DFS in the training set. In the TNBC subtype, cases with high miR-454 expression had a high probability of disease recurrence in univariate analysis (HR = 2.80, 95% CI 1.20-6.56, *P* = 0.018 for training set; HR = 2.50, 95% CI 1.17-5.31, *P* = 0.018 for validation set; Table 3). In multivariate analysis, miR-454 had a significant influence on DFS (HR = 3.81, 95% CI 1.45-10.00, *P* = 0.007 for training set; HR = 3.65, 95% CI 1.52-8.79, *P* = 0.004 for validation set). Moreover, poor condition of axilla and larger tumor size indicated poor DFS. High expression of miR-454 was associated with worse OS in both univariate and multivariate analyses (Table S2 & S3). To summarize, miR-454 is an effective predictive factor of clinical outcome in TNBC.

**Table 2:** Univariate and Multivariate survival analyses of factors associated with disease-free survival in overall population

Variates	Training set				Validation set			
	Univariate		Multivariate		Univariate		Multivariate	
	HR (95%CI)	*P*	HR (95%CI)	*P*	HR (95%CI)	*P*	HR (95%CI)	*P*
Age								
≤50 years	1	-	1	-	1	-	1	-
>50 years	1.00 (0.59-1.72)	0.994	0.93 (0.44-1.96)	0.839	1.01 (0.57-1.79)	0.963	0.76 (0.37-1.56)	0.456
Menstrual status								
Premenopause	1	-	1	-	1	-	1	-
Postmenopause	1.49 (0.86-2.59)	0.155	1.81 (0.80-4.11)	0.156	1.17 (0.66-2.06)	0.597	1.42 (0.70-2.89)	0.334
Grade								
1 or 2	1	-	1	-	1	-	1	-
3	1.49 (0.82-2.72)	0.195	0.99 (0.51-1.91)	0.970	1.10 (0.66-2.06)	0.760	0.77 (0.39-1.51)	0.442
Tumorsize								
≤2 cm	1	-	1	-	1	-	1	-
>2,≤5 cm	1.35 (0.77-2.39)	0.297	1.32 (0.67-2.59)	0.418	1.35 (0.74-2.46)	0.333	1.40 (0.71-2.77)	0.327
>5cm	3.28 (1.13-9.53)	**0.030**	4.02 (1.06-15.31)	**0.041**	1.73 (0.51-5.88)	0.381	1.96 (0.44-8.76)	0.380
Lymph node status								
Negative	1	-	1	-	1	-	1	-
Positive	1.99 (1.15-3.42)	**0.013**	2.26 (1.17-4.35)	**0.015**	1.76 (0.99-3.11)	0.054	2.06 (1.08-3.94)	**0.028**
ER status								
Negative	1	-	1	-	1	-	1	-
Positive	0.74 (0.42-1.32)	0.313	0.51 (0.25-1.05)	0.068	0.71 (0.39-1.31)	0.273	0.65 (0.30-1.42)	0.280
Her-2 status								
Negative	1	-	1	-	1	-	1	-
Positive	0.92 (0.53-1.60)	0.754	0.61 (0.31-1.21)	0.160	0.69 (0.37-1.30)	0.253	0.70 (0.34-1.47)	0.352
miR-454								
Low	1	-	1	-	1	-	1	-
High	2.10 (1.22-3.60)	**0.007**	2.02 (1.10-3.72)	**0.023**	2.08 (1.18-3.69)	**0.012**	2.16 (1.16-4.03)	**0.015**

**Table 3:** Univariate and Multivariate survival analyses of factors associated with disease-free survival in TNBC

Variates	Training set				Validation set			
	Univariate		Multivariate		Univariate		Multivariate	
	HR (95%CI)	*P*	HR (95%CI)	*P*	HR (95%CI)	*P*	HR (95%CI)	*P*
Age								
≤50years	1	-	1	-	1	-	1	-
>50 years	1.06 (0.46-2.45)	0.894	0.54 (0.19-1.54)	0.251	1.10 (0.52-2.31)	0.804	0.76 (0.32-1.85)	0.553
Menstrual status								
Premenopause	1	-	1	-	1	-	1	-
Postmenopause	2.19 (0.89-5.37)	0.087	2.38 (0.72-7.82)	0.155	1.13 (0.54-2.38)	0.743	3.65 (1.52-8.79)	0.437
Grade								
1 or 2	1	-	1	-	1	-	1	-
3	1.11 (0.45-2.72)	0.820	0.59 (0.20-1.71)	0.331	0.92 (0.43-1.99)	0.832	0.74 (0.34-1.61)	0.446
Tumorsize								
≤2 cm	1	-	1	-	1	-	1	-
﹥2,≤5 cm	1.63 (0.63-4.21)	0.312	2.07 (0.66-6.44)	0.211	1.46 (0.65-3.27)	0.360	1.87 (0.77-4.59)	0.169
﹥5cm	6.68 (1.93-23.04)	**0.003**	5.16 (1.12-23.84)	**0.036**	1.13 (0.14-8.97)	0.904	1.65 (0.20-13.72)	0.645
Lymph node status								
Negative	1	-	1	-	1	-	1	-
Positive	2.86 (1.23-6.63)	**0.014**	2.47 (0.83-7.37)	0.105	1.71 (0.81-3.62)	0.161	2.51 (1.06-5.94)	**0.036**
miR-454								
Negative	1	-	1	-	1	-	1	-
Positive	2.80 (1.20-6.56)	**0.018**	3.81 (1.45-10.00)	**0.007**	2.50 (1.17-5.31)	**0.018**	3.65 (1.52-8.79)	**0.004**

### Validation of the relationship of miR-454 with clinical outcome in TCGA and BreastMark databases

The TCGA set included a total of 748 female breast cancer patients, among which 43 cases lacked follow-up data. The median age of the 705 study participants was 57 years (IQR 49-67 years) at the time of surgery, and 53 of 705 cases died after a median follow-up time of 12.13 months (IQR 4.40-38.63 months). Kaplan-Meier analysis showed that negative expression of miR-454 indicated better OS in the overall population (*P* = 0.006, Figure S3) and univariate analysis demonstrated that miR-454 is a risk factor for death (HR = 2.02, 95% CI 1.22-3.34, *P* = 0.007, Table S4). However, this relationship was not statistically significant in subtype analysis. Furthermore, we examined the predictive role of miR-454 in the BreastMark database. In the total population, high expression of miR-454 correlated with worse DFS (*P* = 0.003, Figure S4). These data demonstrate that miR-454 is a powerful prognostic marker in breast cancer.

### Response to chemotherapy according to miR-454 status in TNBC

**Figure 3:**
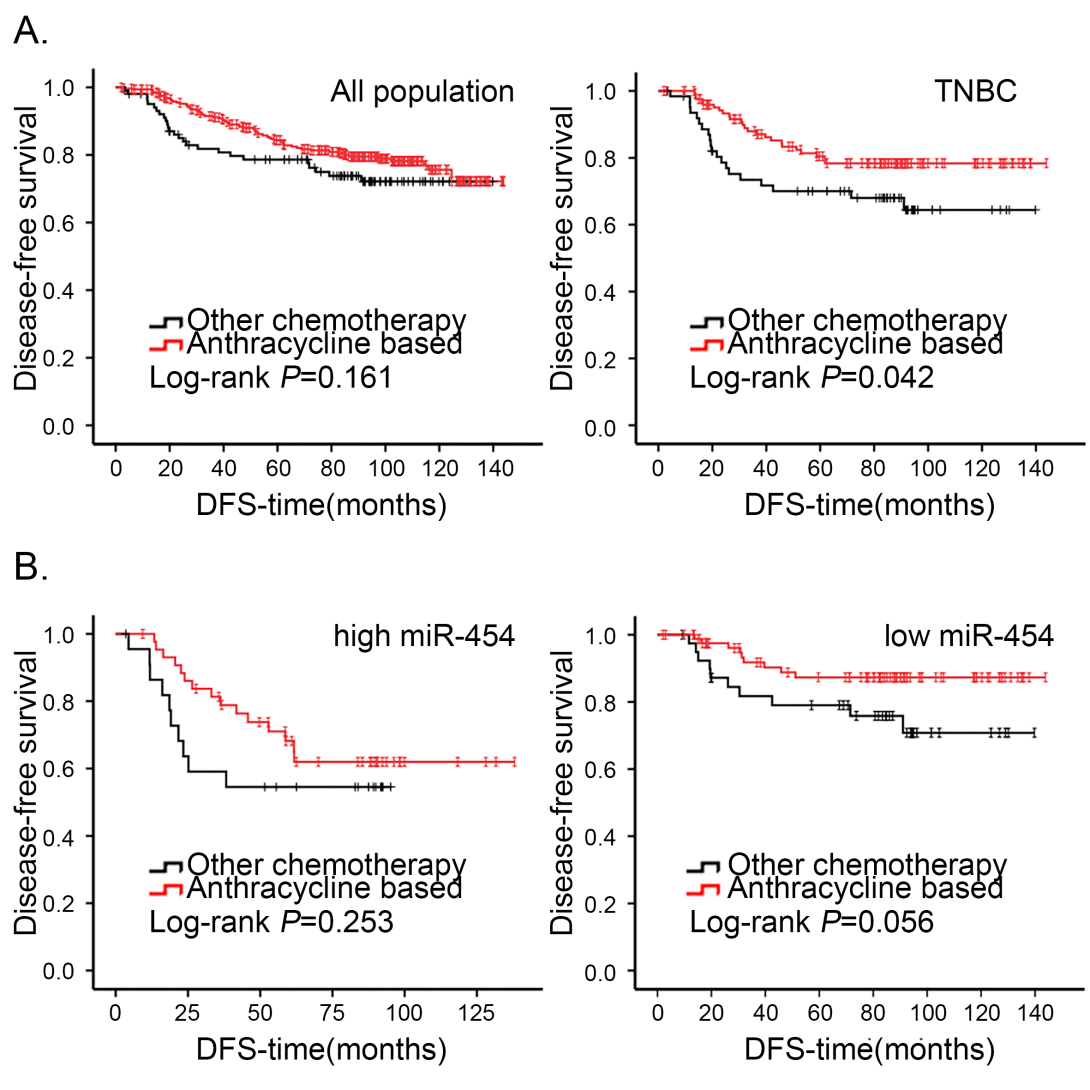
Kaplan-Meier analysis of DFS in patients of breast cancer who received anthracycline-based chemotherapy or other chemotherapy **A.** Cumulative DFS curves of breast cancer patients who received anthracycline-based chemotherapy or other chemotherapy in the overall population and TNBC subtype. **B.** Cumulative DFS curves of breast cancer patients with received anthracycline-based chemotherapy or other chemotherapy in TNBC with miR-454 high or low expression status.

In our cohort, a majority of patients (426/466) had received adjuvant chemotherapy after surgery. Of these, 323 patients had received anthracycline-based chemotherapy (anthracycline plus cyclophosphamide or anthracycline plus cyclophosphamide and 5-fluorouracil) and 43 patients had received taxane-based chemotherapy (single paclitaxel or anthracycline-based chemotherapy followed by or combined with paclitaxel). Some patients received other chemotherapeutic regimens, such as CMF (cyclophosphamide plus methotrexate and 5-fluorouracil). As shown in Figure 3A, there was no statistically significant difference in DFS between patients who received anthracycline-based chemotherapy and those who received non-anthracycline-based chemotherapy for the overall population. However, within the TNBC subtype, the patients who received anthracycline-based chemotherapy had a more favorable DFS (*P* = 0.042, Figure 3A). Because very few patients in the non-TNBC group received non-anthracycline-based chemotherapy, there was no significant difference in DFS according to chemotherapy (data not shown). We next investigated the response to chemotherapy according to expression status of miR-454. In low miR-454 expression patients, patients who had received anthracycline-based chemotherapy had a better clinical outcome than those who had received other chemotherapy (Figure 3B). However, a better response to anthracycline was not observed among high miR-454 expression patients.

## DISCUSSION

miRNAs have been reported to regulate a variety of cellular processes, including tumor progression and metastasis, implying that they might function as biomarkers for diagnosis, prognosis, and therapy response in cancers [19-22]. In previous studies, miR-454 was demonstrated to be a prognostic marker for various types of cancer, such as hepatocellular carcinoma and uveal melanoma. In these studies, miR-454 appeared to function as an oncogene or a tumor suppressor because its expression correlated negatively or positively with tumor biological characteristics and clinical outcome. .

To our knowledge, the relationship between miR-454 and breast cancer clinical outcome has not been published, and our study is the first to evaluate the expression of miR-454 in breast cancer by ISH. Overexpression of miR-454 correlated with worse DFS/OS in the overall breast cancer population, indicating an oncogenic function. Univariate and multivariate survival analyses demonstrated that miR-454 was a predictive factor independent of other traditional prognosis factors, such as tumor size or nodal status. When we considered different breast cancer subtypes stratified by ER and HER2 status, the predictive value of miR-454 for clinical outcome was limited to TNBC. On the basis of our data, we speculated that miR-454 is correlated with malignant biological phenotypes. However, there were more cases of TNBC than other subtypes in our cohort and the predictive role of miR-454 in other subtypes should be explored using more cases and further follow-up.

We also used the publicly available TCGA database to validate our findings. The predictive value of miR-454 for DFS/OS was validated in the overall breast cancer population. However, within the TNBC subtype of the TCGA cohort miR-454 expression was not correlated with OS. We speculated that there might be several reasons for this phenomenon. First, our study population included more TNBC cases and follow-up time was longer than for the TCGA database. Second, the methods used to determine the expression of miR-454 in our study and TCGA are different. To validate our findings, we should add another TNBC validation set or use an additional database.

Anthracycline is an anticancer drug that promotes histone eviction independent of its ability to induce DNA double-strand breaks [23], and the benefit of anthracycline-based therapy in TNBC is supported by several neoadjuvant studies. In our study, the TNBC patients with positive miR-454 status derived less benefit from anthracycline chemotherapy than those without miR-454 expression. Previous research has shown that miRNAs are involved in chemoresistance by impacting target genes or signal pathways [16, 17]. More basic research is required to elucidate the mechanism of resistance to anthracycline-based chemotherapy in the miR-454-positive patients in our study population.

miRNAs inhibit expression of targeted gene by pairing with mRNAs to direct posttranscriptional repression. It has been reported that Smad4 is a direct target of miR-454 in colorectal cancer [6, 24]. Smad4 is an important tumor suppressor involved in TGF-β/BMP signaling[25], and patients with low expression of Smad4 tended to exhibit more poorly differentiated tumors, a higher risk of recurrence and shorter overall survival [26]. Besides, miR-454 was reported to be upregulated in uveal melanoma, functioning as an oncogenic miRNA by targeting PTEN [8]. PTEN, a key negative regulator of PI3K activity, is one of the most frequently mutated suppressor genes in human cancers [27]. PTEN loss in triple-negative breast cancers was significantly associated with poor prognosis [28]. We speculate that miR-454 may represses Smad4 or PTEN expression through direct binding to 3′UTR functioning as an oncogenic miRNA. The hypothesis should be confirmed by further basic research and mechanism of how miR-454 induced poor prognosis by targeted gene need to explore.

There are several limitations of our study. First, our cohort was not representative of the composition of breast cancer because the quantity of each subtype was not equal and there were more cases of TNBC than other subtypes. Second, there are some contradictions in our clinical data as clinicopathological characteristics such as tumor size status were not correlated with DFS in the validation set (Table 2).

In conclusion, we showed that miR-454 is an independent predictor of clinical outcome in breast cancer, especially in the TNBC subtype. In addition, high expression of miR-454 correlated with increased risk of disease events, and miR-454 might be a predictor of therapeutic response in TNBC.

## MATERIALS AND METHODS

### Patients and samples

The present study includes 534 surgical specimens from female patients with stage I-III breast cancer at the Department of Breast Surgery in the Fudan University Shanghai Cancer Center (FDUSCC, Shanghai, P.R. China) between August 2001 and November 2007. Samples were collected in two cohorts: the first cohort (training set) contained 250 cases and the second cohort (validation set) included 284 patients. All patients in the present study underwent mastectomy and axillary lymph node dissection or breast conservation surgery and had histologically confirmed invasive ductal breast carcinoma. All specimens were formalin-fixed and paraffin-embedded (FFPE). Histopathologic information was obtained from the pathology reports and survival data were obtained from telephone or outpatient follow-ups. All clinical characteristics were retrieved and patients were regularly followed up through March 2015, with a median follow-up time of 94.50 and 80.40 months for cohorts 1 and 2, respectively. This study was approved by the Ethics Committee of FDUSCC, and all participants provided written informed consent.

### Tissue microarray and *in situ* hybridization

The method for manufacturing the tissue microarray (TMA) was described previously [29]. 5′-DIG- and 3′-DIG-labeled miRCURY LNA^TM^ Detection probe has-miR-454 (sequence 5′-ACCCTATAAGCAATATTGCACTA-3′) was purchased from EXIQON (Vedbaek, Denmark). *In situ* hybridization kits were purchased from Boster (Wuhan, China). To minimize the decline in TMA quality, TMAs were rewarmed for 4 h at 65°C. TMAs were deparaffinized in xylene solution three times and hydrated sequentially in a gradient of ethanol solutions (100%, 100%, 100%, 95%, 85%, 75%) at room temperature (RT) for 5 min each. To block endogenous peroxidase activity, TMAs were washed with phosphate-buffered saline (PBS) three times and incubated with 3% hydrogen peroxide for 10 min at RT. TMAs were washed with 0.1% DEPC-H_2_O for 5 min, and then incubated with pepsin diluted 10-fold by citrate at 37°C for 20 min to expose the nucleic acid fraction of mRNA. After the digestion procedure, TMAs were washed with PBS three times for 5 min each and with 0.1% DEPC-H_2_O once for 5 min. After incubation with 30 μl pre-hybridization solution for 3 h at 37°C, the TMAs were incubated with 200 μl miRNA probe (20 nM) which had been preheated for 10 min at 80°C and quickly transferred to an ice/water mixture for 5 min, in a hybridization box at 62°C overnight. TMAs were subjected to a stringent washing procedure with 2× saline sodium citrate (SSC, preheated at 37°C, two washes, 5 min each), 0.5×SSC (one wash, 15 min), and 0.2×SSC (one wash, 15 min). After a 30-min wash in blocking solution, TMAs were sequentially incubated with biotinylated digoxin (60 min), streptavidin-biotin complex (SABC, 20 min), and peroxidase (20 min) with a 5-min wash in 0.5M PBS between each. The results were visualized after staining with 3, 3-diaminobenzidine (DAB) and counterstaining with Gill hematoxylin. The U6 probe was used as a positive control following the same procedure.

### Evaluation of miR-454 staining

The staining index (SI), which is a semiquantitative evaluation system incorporating the intensity and percentage of positive cells [30], was used to assess the miR-454 staining results. The staining intensity was classified into four grades: 0, no staining; 1, weak; 2, moderate; 3, strong). The percentage of cells stained was graded as follows: 0, no staining; 1, < 10%; 2, 10-50%; and 3, > 50% tumor cells. SI was calculated by multiplying the grade for percentage staining by the grade for intensity. Each sample was evaluated by two experienced pathologists in a blind manner. Scores of 4 or greater were defined as positive staining and high expression.

### Analysis of the cancer genome atlas (TCGA) database

The TCGA data set [breast invasive carcinoma (BRCA)-IlluminaHiSeq_miRNAseq,*N*= 866] was downloaded from the TCGA data portal (https://tcga-data.nci.nih.gov/tcga/). We used reads per million miRNA as normalized reads of miR-454. After mapping miRNA reads to corresponding clinical cases and excluding the male cases, we defined the median reads as the cutoff.

### Statistical analysis

The significance of differences between variables was evaluated by the Pearson's χ2 test. The Kaplan-Meier method was used to assess DFS/OS, and log-rank test was used to evaluate the difference in survival according to marker status. Patients without events or death were censored at the last follow-up. To determine the correlation of the clinical characteristics with DFS, univariate and multivariate Cox analyses were used to compute hazard ratio (HR) and 95% confidence interval (CI). All date were analyzed by SPSS software (version 22.0; IBM Corporation, Chicago, USA). All P values were two-sided, and a P value less than 0.05 was considered to be statistically significant.

## SUPPLEMENTARY MATERIALS FIGURES AND TABLES










