# Supplementary material for: High expression of microRNA-454 is associated with poor prognosis in triple-negative breast cancer
Source: Oncotarget. 2016 Aug 31;7(40):64900–9. doi: 10.18632/oncotarget.11764 (PMC5323124; doi:10.18632/oncotarget.11764)
Supplement: Supplementary file 1 [file oncotarget-07-64900-s001.pdf]

High expression of microRNA-454 is associated with poor prognosis in triple-negative breast cancer

Supplementary Material

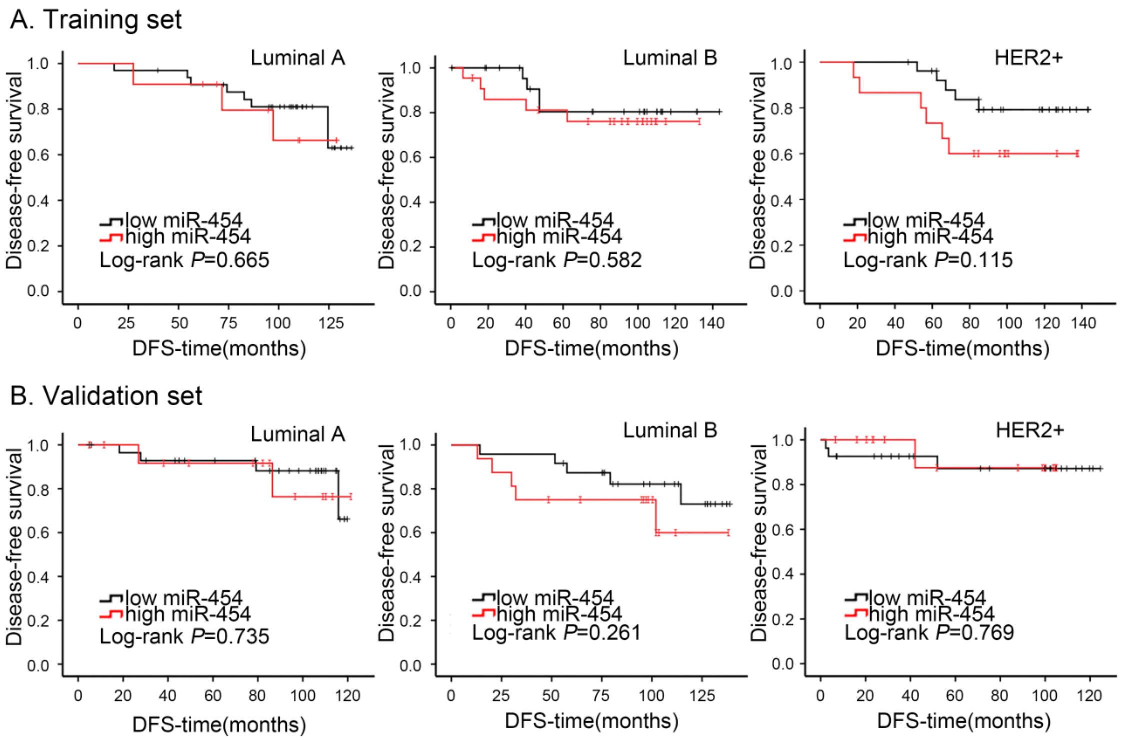

Figure S1. Kaplan–Meier analysis of DFS in patients of breast cancer with high or low miR-454 expression.

A, Cumulative DFS curves of breast cancer patients with high or low miR-454 expression in Luminal A, Luminal B, and HER2+ subtypes of the training set. **B,** Cumulative DFS curves of breast cancer patients with high or low miR-454 expression in Luminal A, Luminal B, and HER2+ subtypes of the validation set.

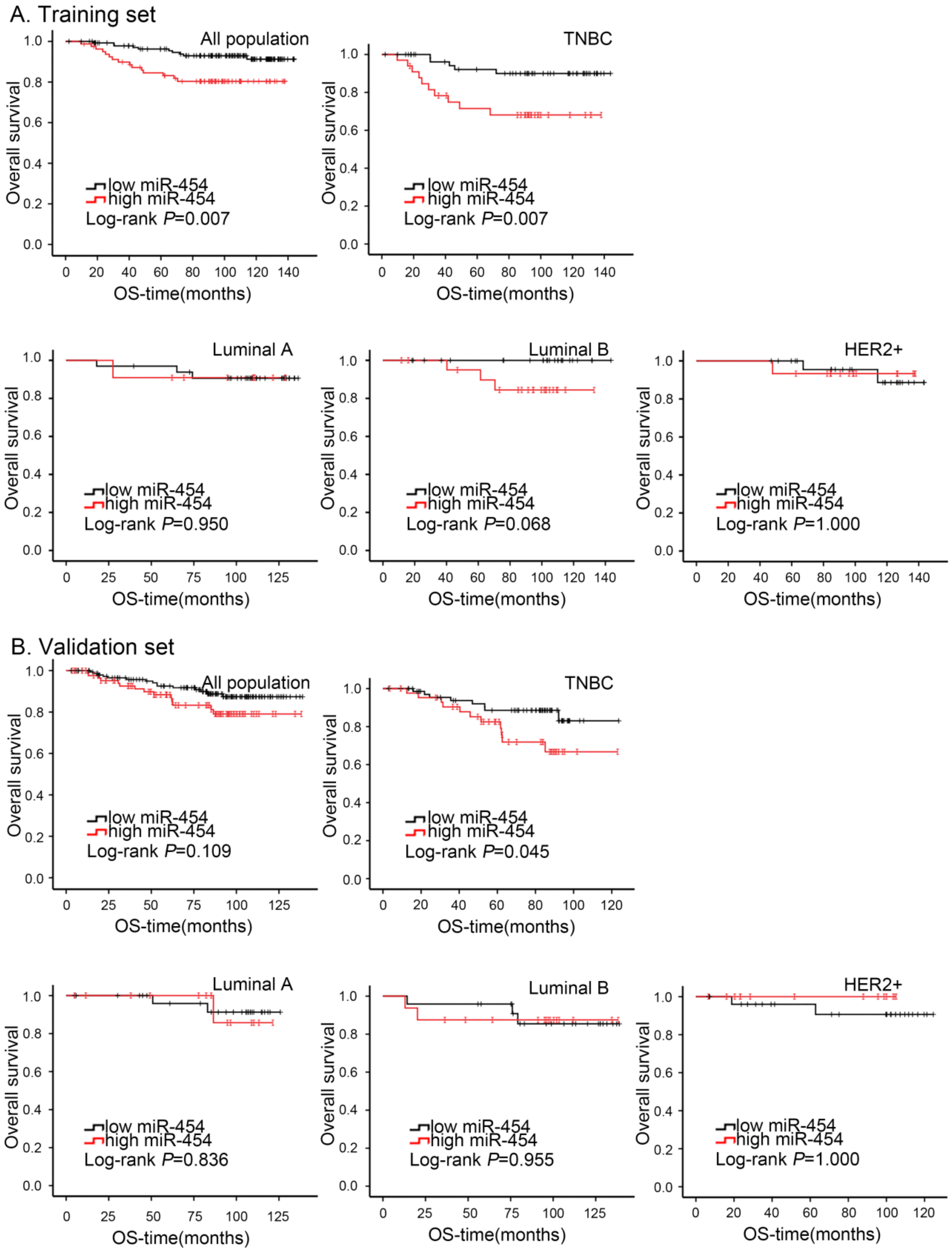

Figure S2. Kaplan–Meier analysis of OS in patients of breast cancer with high or low miR-454 expression.

A, Cumulative OS curves of breast cancer patients with high or low miR-454 expression in overall breast cancer

population and four subtypes of the training set. **B,** Cumulative OS curves of breast cancer patients with high or low miR-454 expression in overall breast cancer population and four subtypes of the validation set.

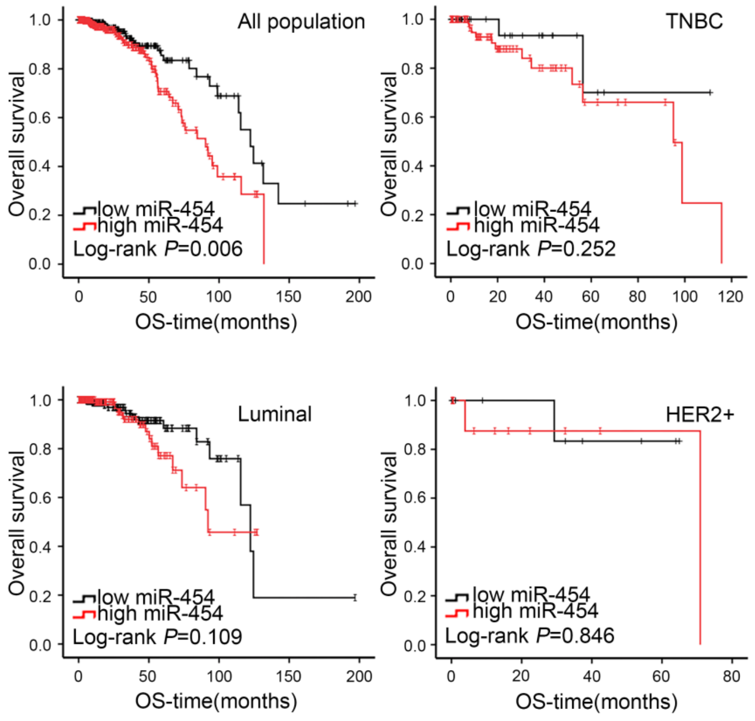

Figure S3. Kaplan–Meier analysis of OS in patients of breast cancer with high or low miR-454 expression in the TCGA data set.

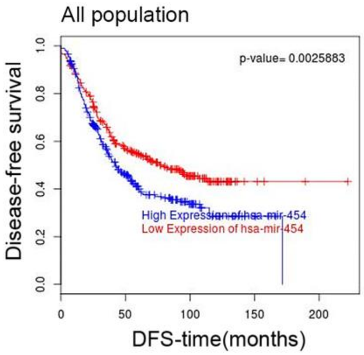

Figure S4. Kaplan–Meier analysis of DFS in patients of breast cancer with high or low miR-454 expression in the BreastMark data set.
